# Visual analysis of emerging topics and trends in contrast agent extravasation research in medical imaging: a bibliometric study using CiteSpace and VOSviewer

**DOI:** 10.3389/fmed.2025.1472637

**Published:** 2025-02-19

**Authors:** Yun Liu, Yonghai Dong, Wenfang Zhou, Juhong Yu

**Affiliations:** ^1^Department of Imaging, Jiangxi Provincial People’s Hospital (The First Affiliated Hospital of Nanchang Medical College), Nanchang, China; ^2^Young Scientific Research and Innovation Team, Jiangxi Provincial Center for Disease Control and Prevention, Nanchang, China

**Keywords:** contrast agent, extravasation, imaging, visual analysis, CiteSpace, VOSviewer

## Abstract

**Purpose:**

This study presents a visualization of the global research dynamics on contrast agent extravasation in medical imaging using a knowledge map, revealing the research directions, emerging topics, trends, and frontiers in this field.

**Method:**

Using CiteSpace and VOSviewer software with the Web of Science Core Collection database as the data source, a bibliometric analysis was conducted on relevant studies of contrast agent extravasation in medical imaging examinations. Analysis was performed on aspects such as yearly publication volume, country/institution distribution, authorship, co-citation documents, and keywords, leading to the creation of visualizations.

**Results:**

A total of 4,635 articles were included in the study, with the first relevant research report appearing in 1950. The yearly publication and citation volumes have shown an overall increasing trend over the years. Research in this field was predominantly concentrated in the United States, accounting for approximately one-third of the global publication output. The University of California System was the top institution in terms of publication volume. The top five high-frequency keywords were “magnetic resonance imaging,” “computed tomography,” “management,” “diagnosis,” and “contrast agent.” Cluster analysis of keywords revealed three main clusters: “contrast,” “fluorescein angiography,” and “focused ultrasound,” showing good continuity over time. The keyword burst analysis identified that “gd dtpa” had the highest burst value of 20.51. The emergence of keywords shifted over time. At present, the keywords that are still emerging are “multimodal imaging,” “case report,” and “leakage.”

**Conclusion:**

More scholars are dedicating efforts to research on contrast agent extravasation in medical imaging. “Multimodal imaging” will be a key research focus in the foreseeable future. Contrast agent extravasation remains a substantial challenge with high research value in medical imaging.

## 1 Background

Contrast agents, also known as contrast media, are predominantly used in medical imaging examinations such as X-ray/CT enhancement, MRI enhancement, and interventional angiography (1–5). Their role is to optimize imaging visualization and create density or signal variations between normal tissue organs and pathological areas for precise diagnostic purposes. Common contrast agents include iodine-based agents, barium-based agents, and MRI contrast agents.

In medical imaging examinations, the use of contrast agents may lead to complications such as allergic reactions (6, 7), vasovagal responses (8), arrhythmias (9), and pulmonary edema (10). The most common complication is contrast agent extravasation (11–13), where the contrast agent (iodine or gadolinium) leaks from the vein into the surrounding soft tissues. Extravasation can result in pain, swelling, blistering, secondary wound infections, tissue adhesions, and even severe outcomes such as compartment syndrome and amputation. An analysis of U.S. data by Dykes et al. (14) showed that the average extravasation rate was 0.24%, and occurrence rates of mild, moderate, and severe injuries due to contrast agent extravasation were 94.6, 4.7, and 0.8%, respectively. These complications not only increase patient discomfort, extend hospital stays, and raise medical costs but also impact physicians’ ability to diagnose, treat, and rescue patients, ultimately affecting patients’ quality of life. To effectively prevent and reduce the occurrence of contrast agent extravasation, researchers need to delve into its mechanisms, influencing factors, and preventive measures. Factors affecting contrast agents include their characteristics (volume, concentration, viscosity, and temperature), injection instruments (various types of catheters), and injection techniques (patient injection sites, injection methods, and infusion rates). By studying the causes of extravasation, clinicians can mitigate adverse factors in clinical practice, improve contrast agents and their administration, and minimize extravasation, thereby reducing potential complications during imaging examinations. However, complete avoidance of contrast agent extravasation is challenging due to high-pressure injections, physical-chemical properties of contrast agent drugs, patient age, underlying diseases, and other factors (15). When extravasation cannot be avoided, targeted measures can be taken for high-risk individuals to reduce the harm caused by extravasation, such as selecting a high-pressure-tolerant central venous catheter for patients with malignant tumors.

The mechanisms of varying degrees of damage caused by contrast agent extravasation primarily include cellular toxicity (16), mechanical compression (17), and osmotic effects (18). *In vitro* studies have suggested that contrast agents can induce morphological changes and apoptosis in endothelial cells. The degree of mechanical compression correlates directly with the amount of extravasation. High-dose contrast agent extravasation can lead to severe rupture of external vein tissues, particularly with high-pressure injections or at hard-to-monitor injection sites. High-osmolarity contrast agents that leak into subcutaneous tissues increase local tissue osmotic pressure, leading to endothelial cell dehydration and local platelet aggregation, resulting in changes such as increased capillary permeability, leukocyte infiltration, and release of inflammatory chemoattractants such as prostaglandins E1 and E2, causing inflammation (19).

As medical imaging technology continues to advance, new types of contrast agents will emerge, necessitating continuous updating and enhancement of research on contrast agent extravasation. This study aimed to systematically review and analyze the research hotspots and development trends of contrast agent extravasation in medical imaging examinations through visual methods. We strived to provide valuable reference information for researchers and practitioners in related fields, offering safer and more effective technical support for clinical practice in medical imaging.

## 2 Materials and methods

### 2.1 Inclusion and exclusion criteria

The Web of Science (WOS) Core Collection was utilized as the search database, including citation indices such as the Science Citation Index Expanded, Social Sciences Citation Index, and Emerging Sources Citation Index. The included literature had to meet the following criteria: (1) studies related to contrast agent extravasation following intravenous injection in medical imaging examinations and (2) publication dates ranging from the establishment of the database to 31 December 2024. The exclusion criteria for the literature comprised (1) meeting abstracts, (2) letters, (3) editorial materials, (4) corrections, (5) proceedings, (6) book chapters, (7) notes, and (8) duplicate publications.

### 2.2 Literature search

**Figure 1:**
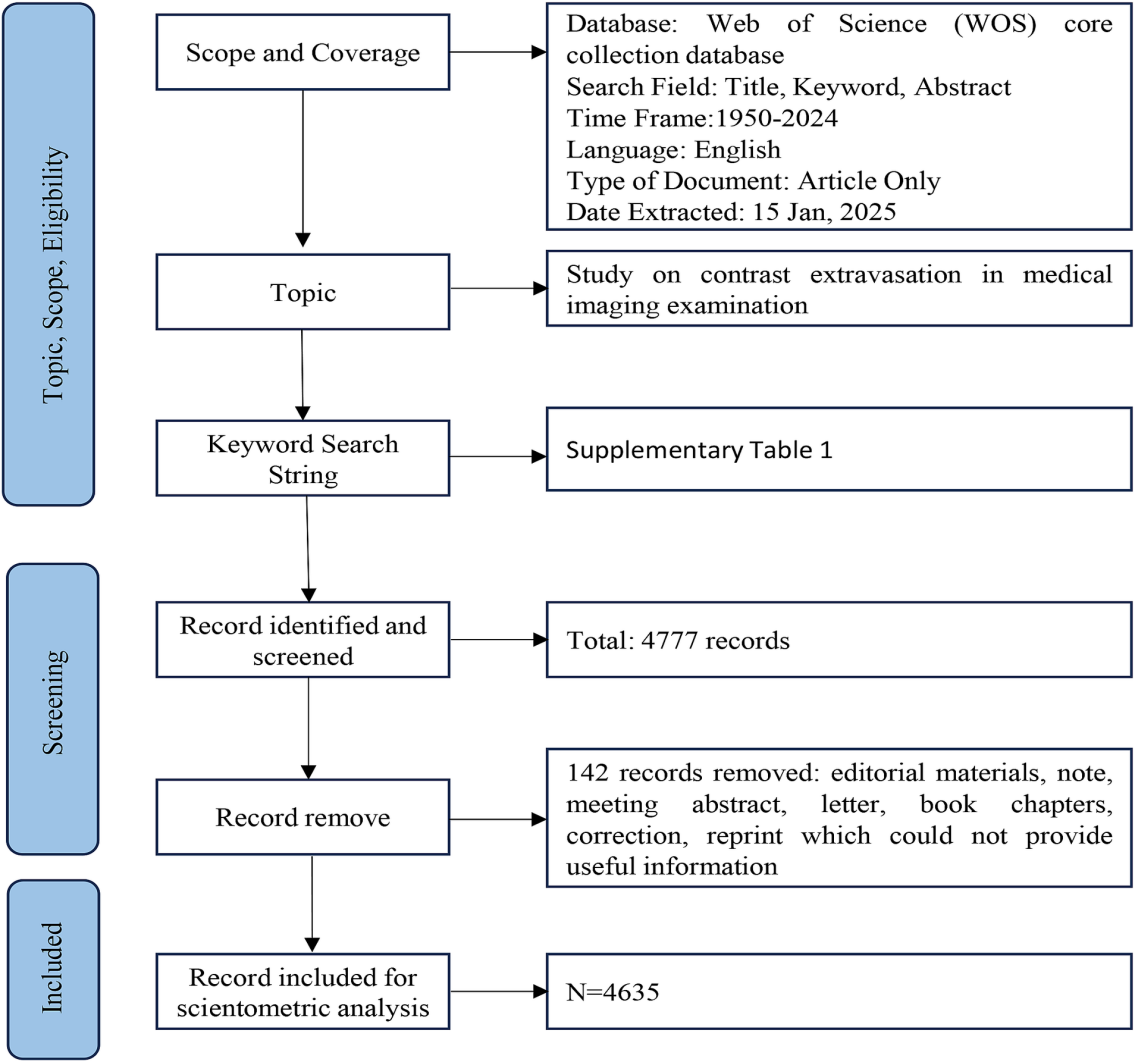
The research flowchart.

The specific search strategies used in the Web of Science (WOS) Core Collection are outlined in Supplementary Table S1. The search duration extended from the establishment of the database to 31 December 2024. A total of 4,777 relevant publications were identified, with 142 publications, such as meeting abstracts, letters, editorial materials, corrections, proceedings, notes, and book chapters, excluded due to the lack of essential information for bibliometric analysis. Using CiteSpace, a duplicate check was conducted, and no duplicate literature was found. Finally, 4,635 publications were included, and the literature search and selection process are detailed in Figure 1.

### 2.3 Statistical analysis

Statistical analysis was conducted using CiteSpace 6.2, VOSviewer 1.6, and Excel 2016 software to identify journals, institutions, countries, keyword co-occurrence, co-cited articles, and trends. These software tools were also used to visualize the knowledge map.

The analysis of keyword/topic co-occurrence was used to identify research hotspots within specific fields. Co-citation analysis and keyword burst examination were used to determine the research frontiers in the field. Clustering analysis of keywords/topics, coupled with timeline graph analysis, was undertaken to trace the evolutionary path of research. Exploring author/institutional collaboration networks helps in identifying notable figures and core groups within the research domain. Furthermore, co-occurrence network analysis between journals facilitates interdisciplinary integration across fields of study.

## 3 Results

### 3.1 Analysis of annual publication volume

**Figure 2:**
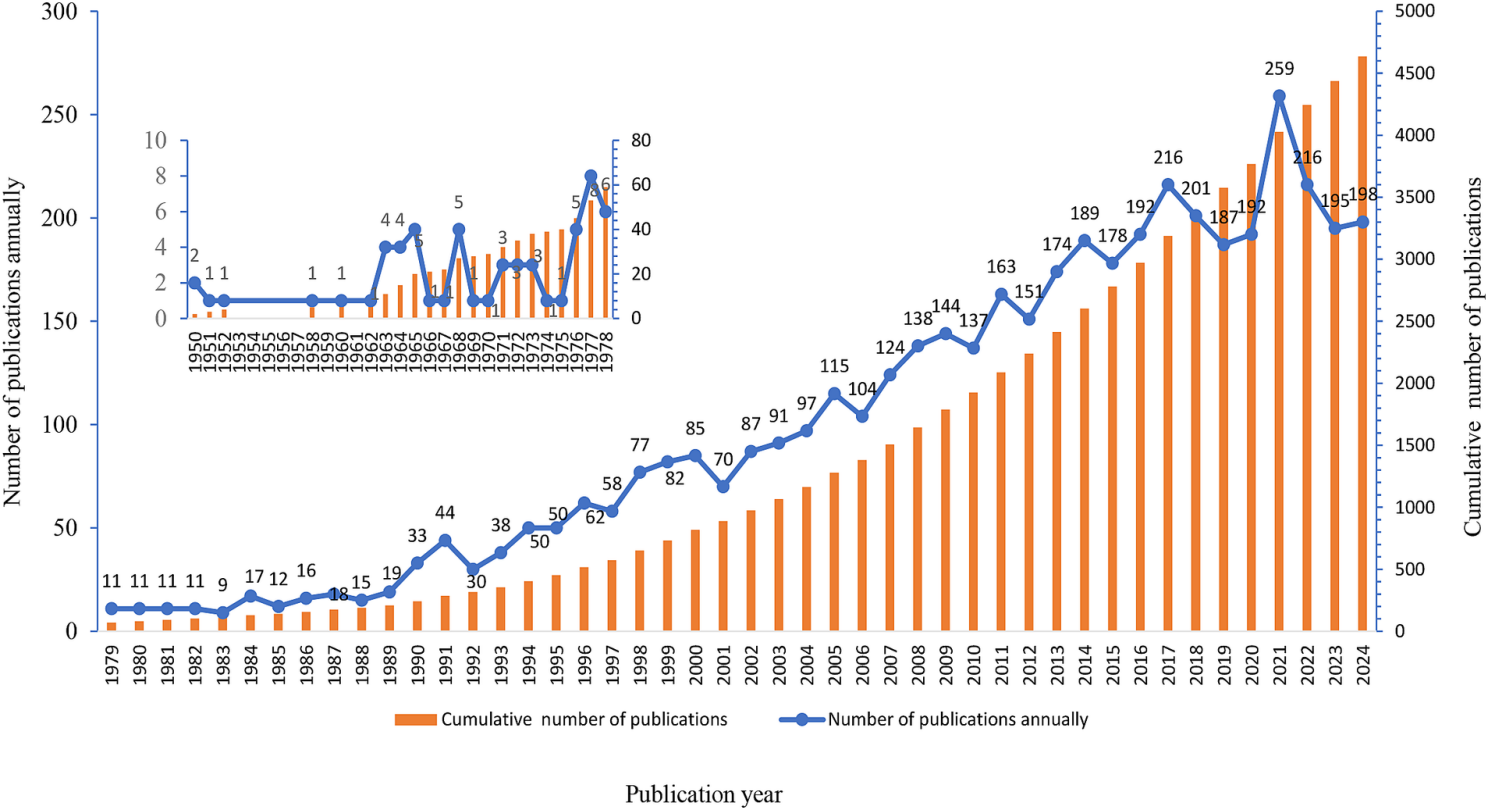
Annual and cumulative trend of publications.

A total of 4,635 valid articles were included in this study, with the first research paper in the field published in 1950 (20). Between 1950 and 2024, the volume of relevant research publications in this field has been consistently increasing year by year (Figure 2).

### 3.2 Analysis of national, institutional, and author collaboration network

#### 3.2.1 Analysis of national publication volume and co-occurrence

**Figure 3:**
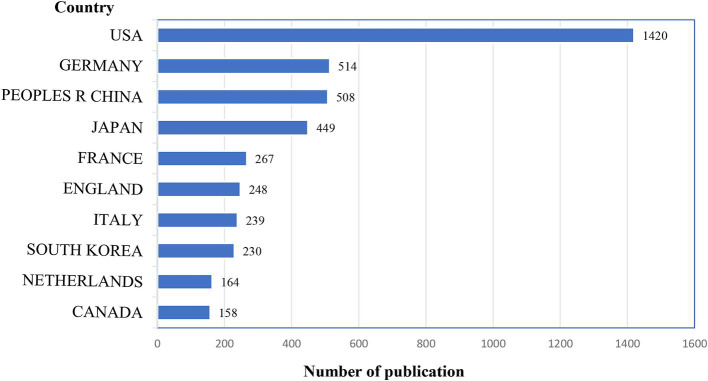
The distribution of the top 10 countries that had the majority of publications.

**Figure 4:**
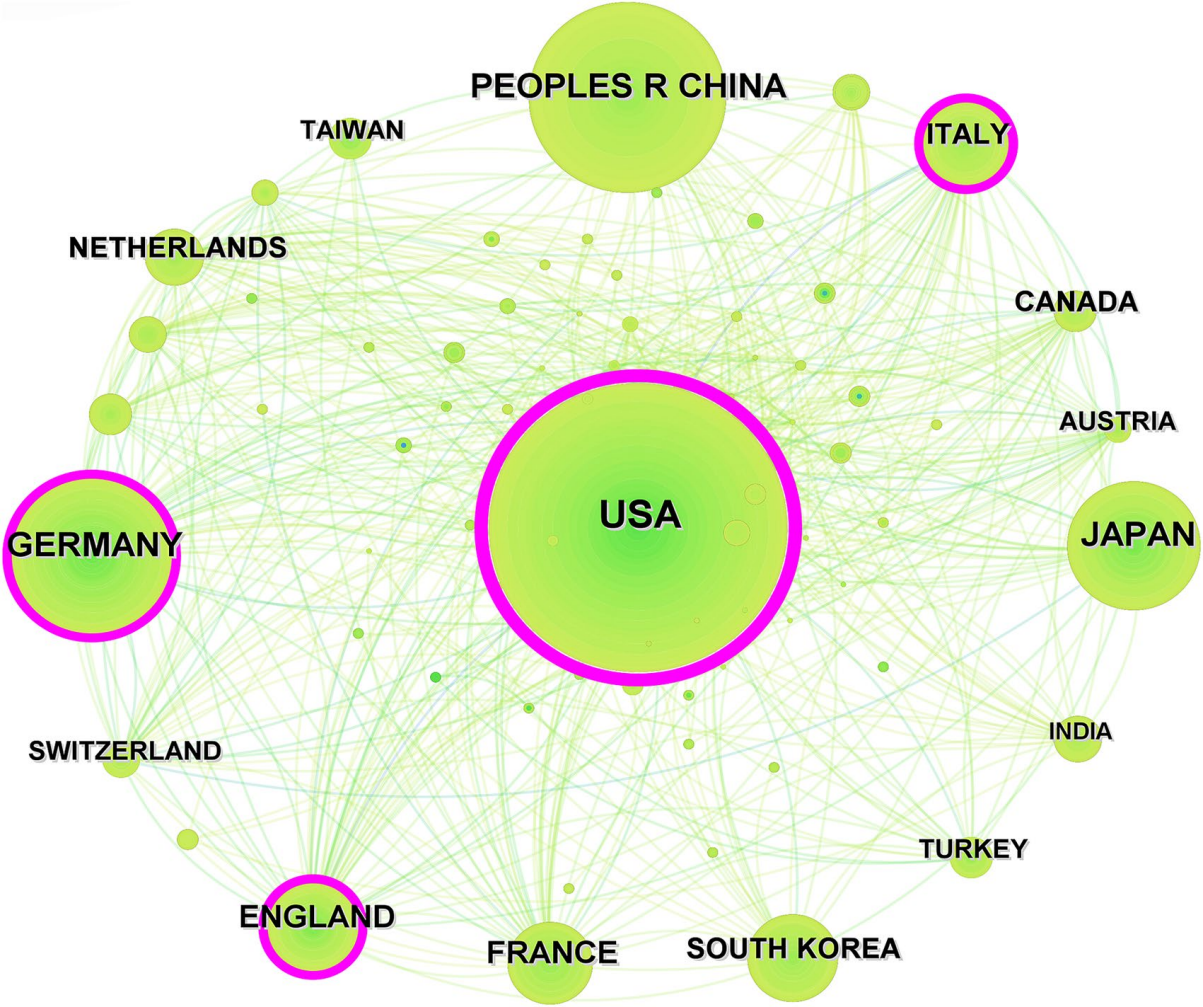
Countries co-occurrence analysis.

The United States emerged as the leading country in terms of publication volume, contributing to one-third of the total publications with 1,420 articles, followed by Germany (514 articles), and China (508 articles). Figure 3 displays the top 10 countries by publication count. In this research domain, the high intermediary centrality was predominantly observed in the United States (0.50), England (0.14), Italy (0.13), and Germany (0.11), while intermediary centrality in other countries was below 0.10, as shown in Figure 4.

#### 3.2.2 Analysis of institutional publication volume and collaboration network

**Figure 5:**
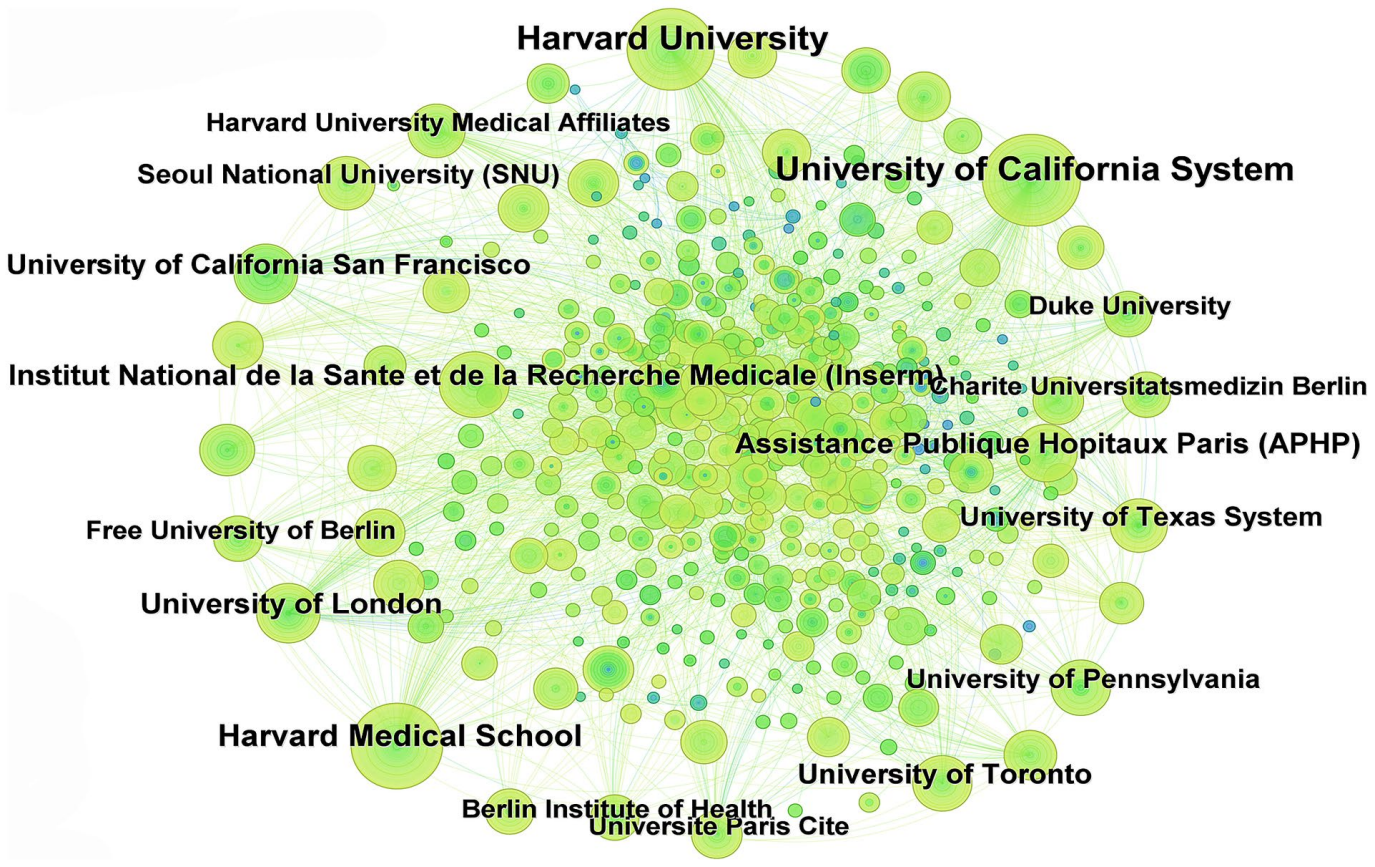
Cooperative network diagram of research institutions.

In this field, a total of 12,777 institutions are involved. Figure 5 illustrates the collaboration network of research institutions in this field, where nodes represent institutions; the larger the node, the greater the publication output of the institution. Thicker lines between nodes indicate stronger collaboration intensity. The top 10 institutions in terms of publication volume are detailed in Table 1. The top three research institutions in terms of publication output are the University of California System (169 articles), Harvard University (150 articles), and Harvard Medical School (100 articles). Only one institution has intermediary centrality in collaboration exceeding 0.1, namely the University of California System (0.17).

**Table 1:** Top 10 institutions in terms of number of publications and intermediary centrality.

Rank	Publication		Centrality	
	Number of publications	Institution	Centrality	Institution
1	169	University of California System	0.17	University of California System
2	150	Harvard University	0.09	Harvard University
3	100	Harvard Medical School	0.06	University of London
4	68	Institut National de la Sante et de la Recherche Medicale (Inserm)	0.05	Institut National de la Sante et de la Recherche Medicale (Inserm)
5	66	University of London	0.05	Assistance Publique Hopitaux Paris (APHP)
6	65	Assistance Publique Hopitaux Paris (APHP)	0.04	Harvard Medical School
7	59	University of Toronto	0.04	Centre National de la Recherche Scientifique (CNRS)
8	55	University of California San Francisco	0.04	Helmholtz Association
9	49	University of Pennsylvania	0.03	University of Toronto
10	48	University of Texas System	0.03	University of Texas System

#### 3.2.3 Analysis of author collaboration network and co-cited authors

**Figure 6:**
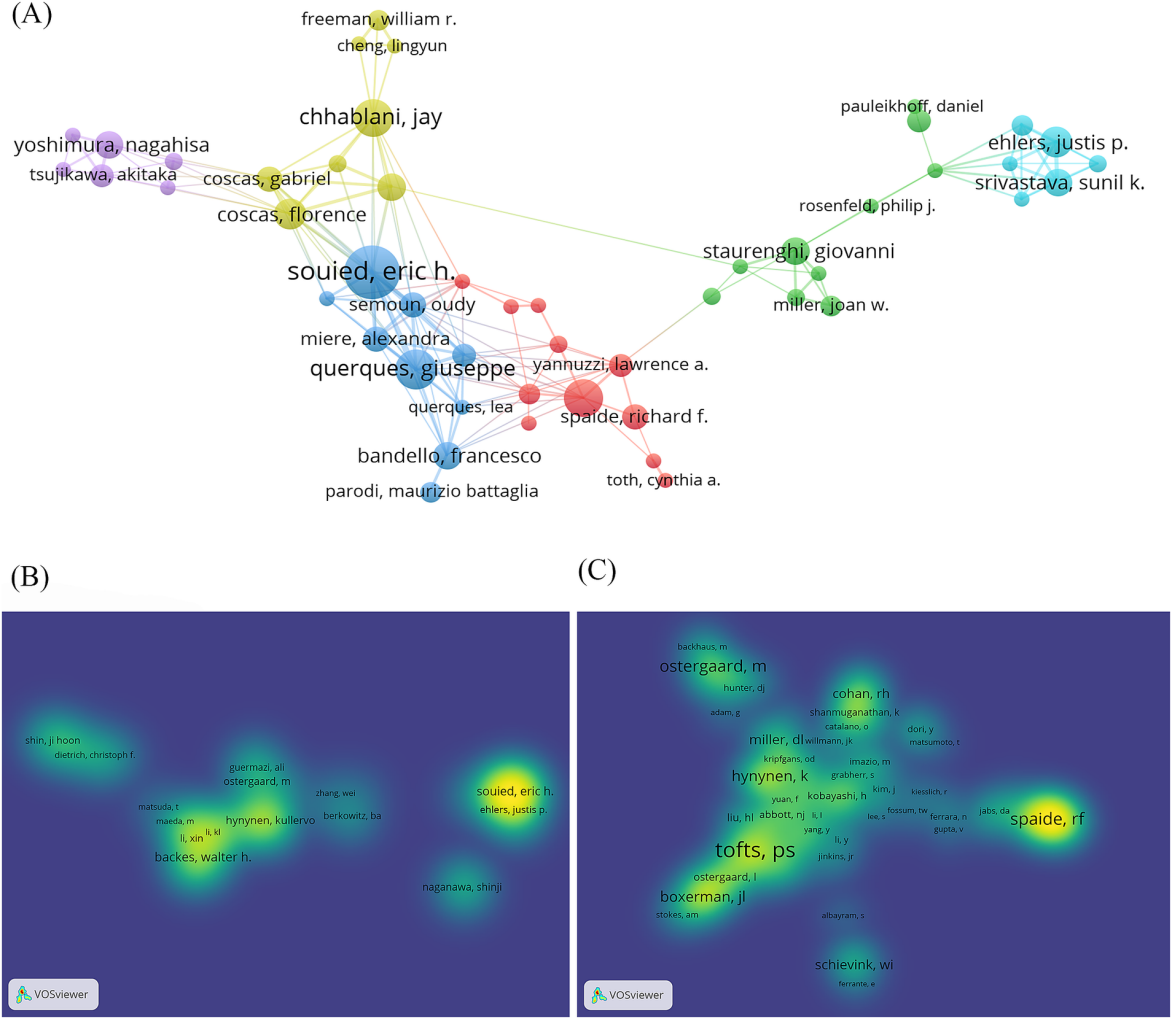
Author cooperation network map. **(A)** Network visualization of co-authorship of the authors. **(B)** Density visualization of citation of authors. **(C)** Density visualization of co-citation of authors.

Among the 4,635 retrieved documents, a total of 23,919 authors are represented. To identify influential authors in this field, Figure 6 illustrates the collaboration network among all authors and co-cited authors. Figure 6A shows the co-authorship of authors with more than two appearances, while Figures 6B,C illustrate the collaboration depth of 231 cited authors with at least five publications and 532 co-cited authors with a minimum of 20 citations, respectively.

Table 2 presents the top 10 authors by publication volume and the top 10 authors by co-citation frequency. The analysis reveals the top three authors by publication volume as Backes W. H. (23 articles), Souied E. H. (21 articles), and Quarles C. C. (16 articles), while the top three authors by co-citation frequency are Tofts P. S. (472 times), Spaide R. F. (302 times), and Ostergaard M. (282 times). These findings indicate widespread recognition of the research contributions of these scholars in this field.

**Table 2:** Top 10 authors and co-cited authors in the studies.

Rank	Authors		Co-cited authors	
	Author	Publications (*n*)	Co-cited author	Co-citations (*n*)
1	Backes W. H.	23	Tofts P. S.	472
2	Souied E. H.	21	Spaide R. F.	302
3	Quarles C. C.	16	Ostergaard M.	282
4	Hynynen K.	16	Hynynen K.	232
5	Jansen J. F. A.	15	Yannuzzi L. A.	218
6	Querques G.	15	Boxerman J. L.	211
7	Freund K. B.	14	Mcdannold N.	187
8	Chhablani J.	14	Schievink W. I.	186
9	Schmidt-erfurth U.	14	Miller D. L.	171
10	Ostergaard M.	14	Gass J. D. M.	171

### 3.3 Analysis of co-cited literature network

Table 3 presents the top 10 most frequently cited papers (21–30). The most cited paper is “Quantitative optical coherence tomography angiography of choroidal neovascularization in age-related macular degeneration” by Jia et al. (26) in 2014, with a total of 26 citations. This is followed by the paper “Estimating kinetic parameters from dynamic contrast-enhanced T_1_-weighted MRI of a diffusable tracer: Standardized quantities and symbols” published by Tofts et al. (22), with a total of 256 citations.

**Table 3:** Top 10 most cited research articles.

ID	Title	Journal	Publication year	First author	Citations (*n*)
1	Quantitative optical coherence tomography angiography of choroidal neovascularization in age-related macular degeneration	Ophthalmology	2014	Jia Y. L.	26
2	Estimating kinetic parameters from dynamic contrast-enhanced T_1_-weighted MRI of a diffusable tracer: standardized quantities and symbols	J Magn Reson Imaging	1999	Tofts P. S.	25
3	Spectral-domain optical coherence tomography angiography of choroidal neovascularization	Ophthalmology	2015	de Carlo T. E.	23
4	Blood-brain barrier breakdown in the aging human hippocampus	Neuron	2015	Montagne A.	22
5	Retinal vascular layers imaged by fluorescein angiography and optical coherence tomography angiography	JAMA Ophthalmol	2015	Spaide R. F.	21
6	Tracer kinetic modeling for DCE-MRI quantification of subtle blood-brain barrier permeability	NeuroImage	2016	Heye A. K.	19
7	Optical coherence tomography angiography of type 1 neovascularization in age-related macular degeneration	Am J Ophthalmol	2015	Kuehlewein L.	18
8	Quantifying blood-brain barrier leakage in small vessel disease: Review and consensus recommendations	Alzheimers Dement	2019	Thrippleton M. J.	18
9	Relative cerebral blood volume maps corrected for contrast agent extravasation significantly correlate with glioma tumor grade, whereas uncorrected maps do not	Am J Neuroradiol	2006	Boxerman J. L.	18
10	Quantification of endothelial permeability, leakage space, and tumor blood volume in a rat brain tumor model using dynamic susceptibility contrast-enhanced MRI	J Magn Reson Imaging	2000	Zhu X. P.	17

### 3.4 Keyword analysis

#### 3.4.1 Co-occurrence analysis

**Figure 7:**
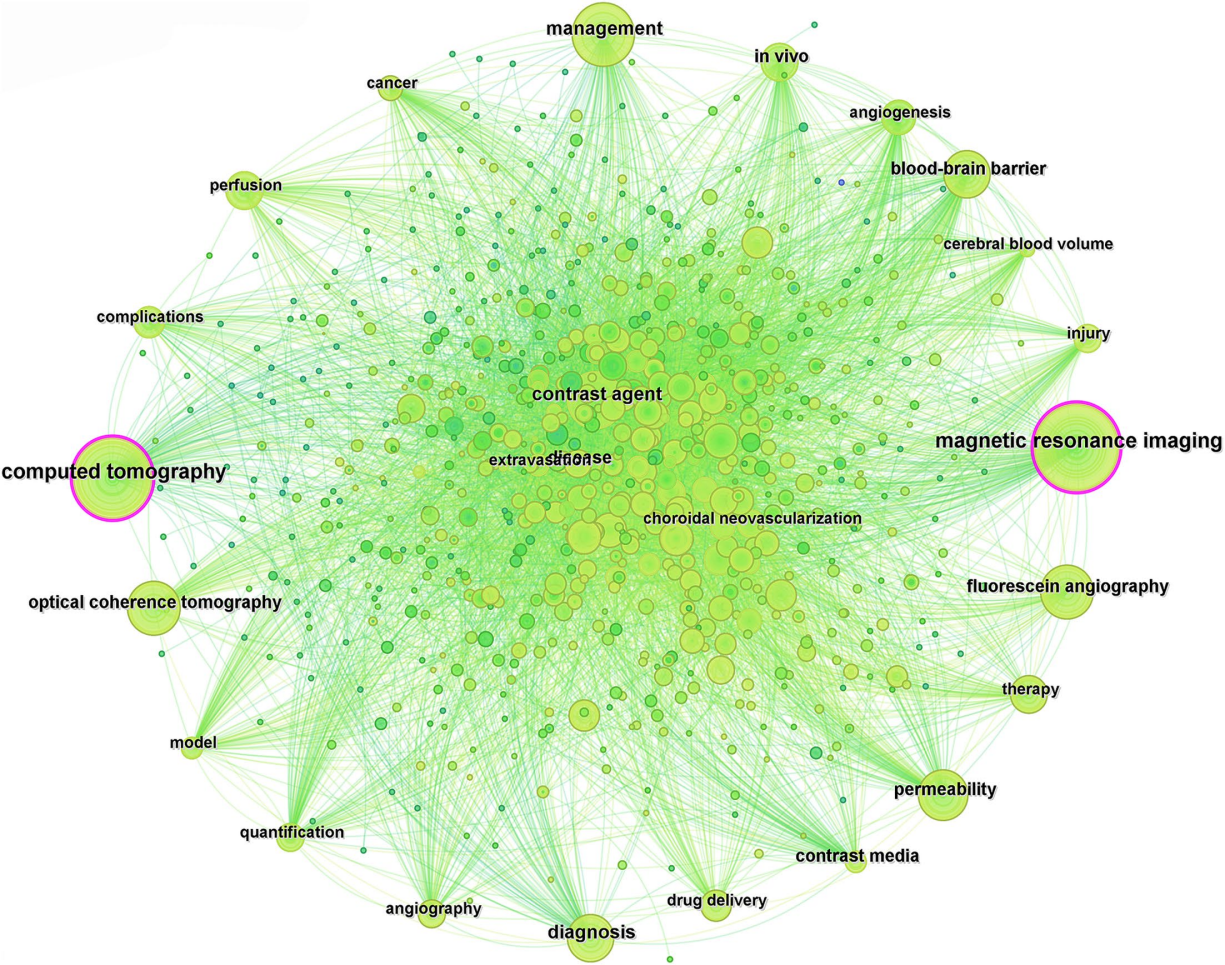
Keyword contribution analysis.

Keywords serve as concise summaries of the central themes and crucial information in the literature. By summarizing and dissecting the keywords in documents, it becomes possible to explore the research trends and directions within a particular field. Using CiteSpace for co-occurrence analysis of the keywords in the included literature, equivalent terms were consolidated, such as “contrast agent” and “contrast agents,” “agent” and “agents,” “magnetic resonance imaging,” and “mri,” and “computed tomography” and “ct,” resulting in a total of 794 related keywords (Figure 7). The top 20 high-frequency keywords in terms of occurrence are listed in Table 4, with the top five keywords being “magnetic resonance imaging,” “computed tomography,” “management,” “diagnosis,” and “contrast agent.”

**Table 4:** Top 20 high-frequency keywords.

Rank	Count	Keyword	Rank	Count	Keyword
1	468	magnetic resonance imaging	11	152	contrast media
2	443	computed tomography	12	151	disease
3	366	management	13	130	therapy
4	288	diagnosis	14	130	injury
5	254	contrast agent	15	125	complications
6	201	permeability	16	125	drug delivery
7	199	optical coherence tomography	17	122	perfusion
8	181	fluorescein angiography	18	118	choroidal neovascularization
9	170	blood-brain barrier	19	116	angiography
10	169	*in vivo*	20	112	cancer

#### 3.4.2 Cluster analysis

**Figure 8:**
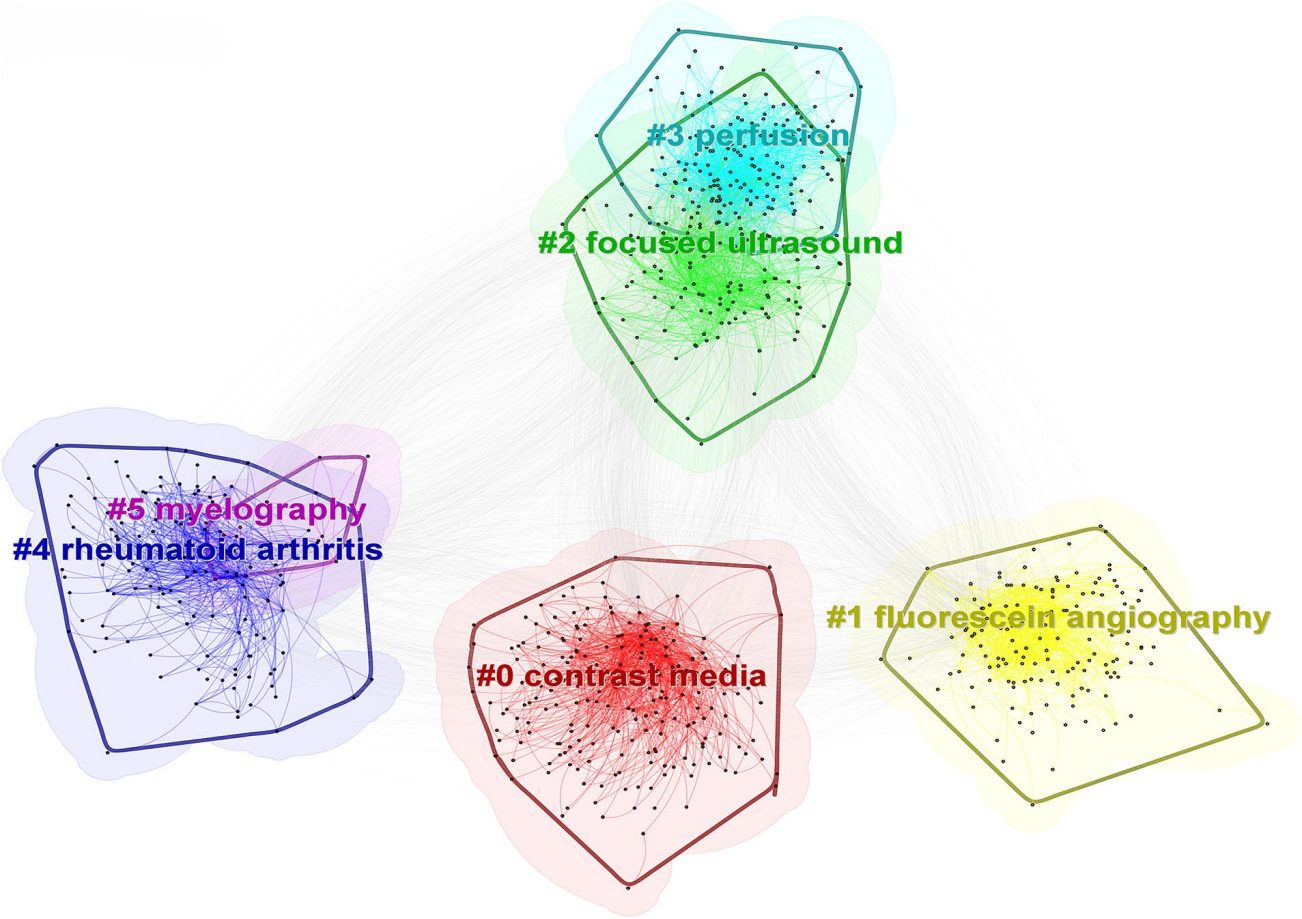
Network map of keywords.

Cluster analysis of keywords revealed that the keywords primarily formed six clusters, namely “contrast media,” “fluorescein angiography,” “focused ultrasound,” “perfusion,” “rheumatoid arthritis,” and “myelography.” The clusters of “focused ultrasound” and “perfusion,” as well as “rheumatoid arthritis” and “myelography,” exhibit overlaps, indicating interdisciplinary integration in this particular research domain (Figure 8).

#### 3.4.3 Timeline and emergent keyword analysis

**Figure 9:**
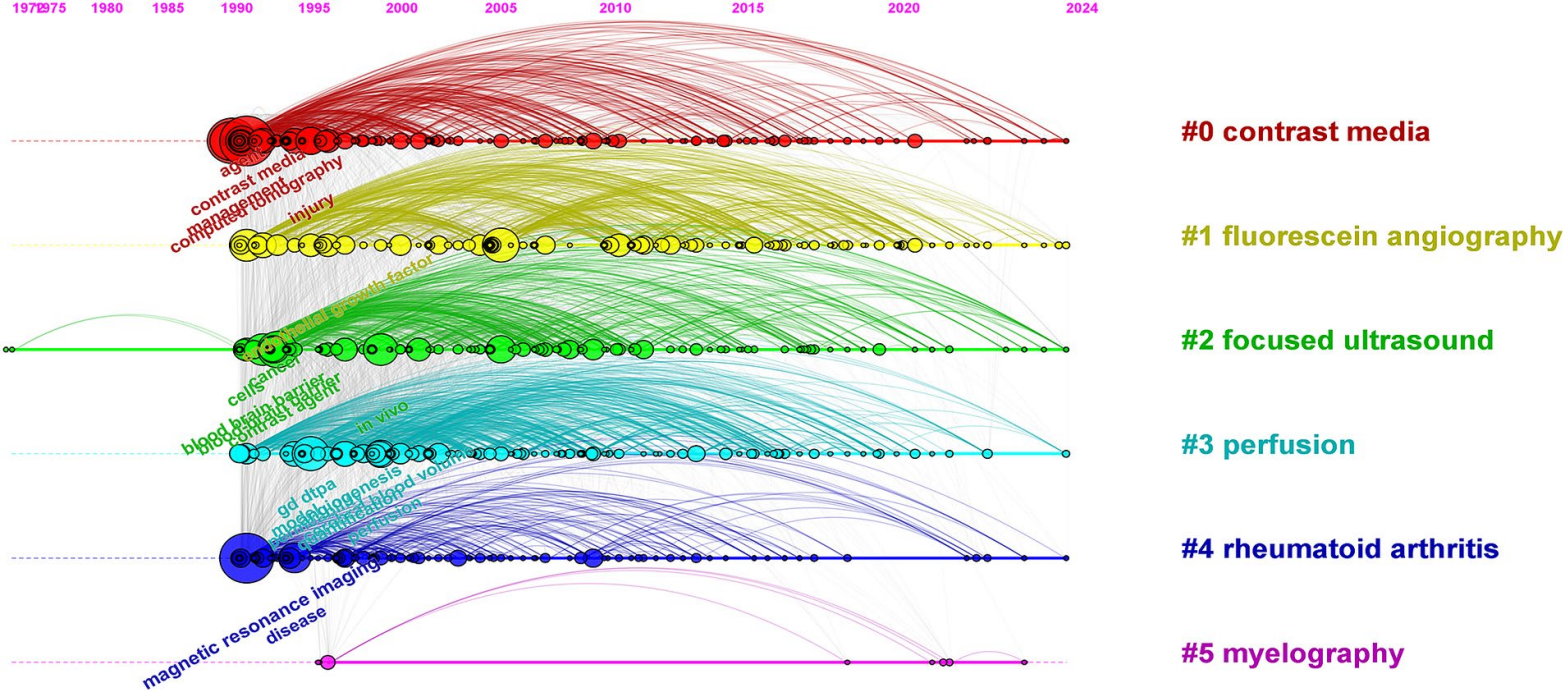
Keyword timeline distribution.

CiteSpace’s timeline spectrum graph enables a visual representation of the evolving hotspots and their interconnected relationships in different research areas over time, facilitating predictions for future developments. Based on the literature from the WOS Core Collection database, the timeline spectrum of keyword clusters in the research was obtained, as shown in Figure 9. Notably, the clusters of keywords “contrast media,” “fluorescein angiography,” “focused ultrasound,” and “perfusion” exhibit a significant continuity in their temporal progression.

**Figure 10:**
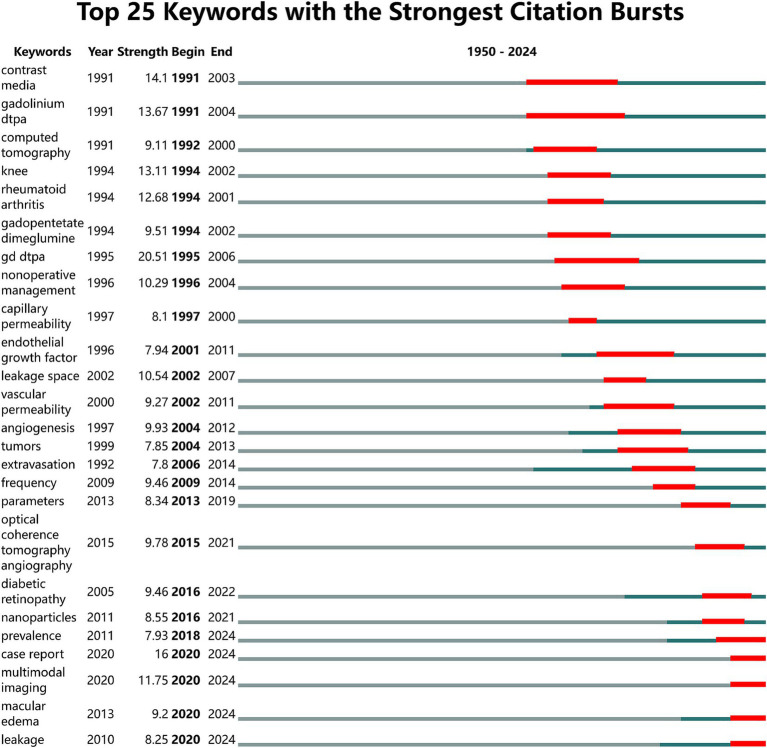
Map of top 25 keywords with the strongest citation bursts. The red line segments represent the time periods of keyword emergence, with the positions at both ends indicating the corresponding starting year (begin) and ending year (end). The green line segments represent the remaining years outside the time periods of keyword emergence.

CiteSpace’s burst detection, known as emergent keyword analysis, enables the analysis of the research frontiers, evolving hotspots, and development trends in academic domains. The main graph of emergent keywords in the study includes information on emergent keywords, emergence strength, and start and end times. Figure 10 presents the top 25 key emergent terms in research on contrast agent extravasation in medical imaging examinations. In the emergence graph, “Strength” represents the emergence value, where a higher value indicates a higher credibility of the term’s emergence in a specific period. In our study literature, “gd dtpa” had the highest emergence value of 20.51. Analysis of the annual fluctuations in emergent terms revealed that from 1991 to 2001, the main emergent terms included “contrast media,” “gd dtpa,” and “computed tomography,” persisting for over 10 years. From 2001 to 2014, the emergent terms gradually shifted to “endothelial growth factor,” “vascular permeability,” “angiogenesis,” and “tumors.” From 2014 to 2024, the terms “multimodal imaging,” “macular edema,” “case report,” and “leakage” reemerged, remaining in the emergent phase.

## 4 Discussion

This study is the first to apply bibliometric methods, using visual analysis tools such as CiteSpace and VOSviewer, to comprehensively showcase the global dynamics of research on contrast agent extravasation in medical imaging. It vividly illustrates the trends, current status, research hotspots, and emerging frontiers over the past seven decades.

An analysis of the annual publication numbers revealed that the initial study reported in the WOS Core Collection database emerged in 1950 (20). However, since 1979, the number of publications has increased annually, which is closely associated with the rapid development and widespread adoption of medical imaging technologies such as ultrasound, CT, and MRI (31, 32). With the extensive use of advanced medical imaging technologies, the role of contrast agents in medical imaging examinations has become increasingly prominent, shedding light on the issue of extravasation of contrast agents. During this period, researchers not only continued to focus on contrast agent enhancement techniques but also delved deeper into exploring the mechanisms, prevention, and treatment methods of contrast agent extravasation, thereby propelling rapid progress in this research field (33).

In terms of the number of publications by country and co-occurrence analysis results, the United States leads significantly with 1,420 publications, representing one-third of the total global publication output, followed by Germany and China. In this research field, high intermediation centrality is primarily concentrated in European and American countries, indicating the substantial international influence of these nations. Among the 12,777 relevant research institutions worldwide, the University of California System (169 articles) and Harvard University (150 articles) stand out as the institutions with the highest publication volume, extensive collaborative networks, and significant impact. Analysis of author collaboration networks revealed the emergence of several large-scale networks of scholars driving advancements in the research field of extravasation of contrast agents in medical imaging.

Keywords serve as concise summaries of the main themes and essential information in literature, enabling the exploration of research hotspots and directions in a specific field. Analysis of keyword co-occurrence in this field revealed that “magnetic resonance imaging,” “computed tomography,” “management,” “diagnosis,” and “contrast agent” are the top five keywords with the highest frequency of occurrence. Co-occurrence analysis and clustering uncovered the research hotspots and trends in the fields of medical imaging and drug delivery (34). The top three clusters, namely, “contrast media,” “fluorescein angiography,” and “focused ultrasound,” highlight the significance and activity of these technologies in medical diagnosis and treatment. Research findings indicated that there is an overlap between the clusters of “focused ultrasound” and “perfusion,” as well as between “rheumatoid arthritis” and “myelography.” This suggests a profound interdisciplinary integration that facilitates the generation of innovative outcomes. This high level of integration signifies that interdisciplinary research is propelling these fields toward more comprehensive and in-depth developments. Furthermore, the continuity over time of the clusters “contrast media,” “fluorescein angiography,” “focused ultrasound,” and “perfusion” indicates that these studies are not only current hotspots but are likely to maintain their research vitality and depth in the future, offering more innovative solutions and treatment strategies for clinical medicine.

Among the numerous keywords the term “gd dtpa” had the highest burst value of 20.51 indicating it is a highly representative research topic that has garnered widespread attention in imaging practices and become a focal point for researchers (35–37). The burst keywords in this field evolve continually. Before 2001 research primarily focused on the preliminary applications of contrast agents and their integration with imaging technologies. Keywords such as “contrast media,” “gadolinium dtpa,” and “computed tomography” indicate that researchers began to explore the application of contrast agents in imaging examinations such as CT to enhance image quality and diagnostic accuracy. Simultaneously the emergence of terms such as “capillary permeability” and “leakage space” signifies increasing attention to the phenomenon of contrast agent extravasation as researchers sought to understand the underlying mechanisms. From 2001 to 2014 the research focus shifted toward investigating the mechanisms of contrast agent extravasation and exploring its clinical significance. Keywords such as “endothelial growth factor,” “angiogenesis,” and “vascular permeability” illustrate researchers’ efforts to examine the mechanisms of extravasation at the cellular and molecular levels particularly regarding factors related to endothelial cells. In addition the emergence of terms such as “tumors” and “extravasation” indicates that the clinical relevance of contrast agent extravasation in diseases such as tumors gained attention with researchers attempting to evaluate disease progression and treatment efficacy through this phenomenon. From 2014 to the present research has concentrated on the application of multimodal imaging techniques and the comprehensive management of contrast agent extravasation. Keywords such as “optical coherence tomography,” “nanoparticles,” and “multimodal imaging” indicate that researchers started integrating various imaging technologies with emerging fields such as nanotechnology to more comprehensively assess contrast agent extravasation. Furthermore terms such as “prevalence,” “case report,” and “macular edema” reflect an increased emphasis on the prevalence of contrast agent extravasation clinical case analyses and potential complications indicating a growing focus on the clinical applications and risk assessments associated with the extravasation phenomena. It is important to note that the emerging significance of “multimodal imaging” is considerably high at this stage indicating that it may become a focal point of research in the near future (5, 38–40). The burst keyword analysis not only highlights the growth of interdisciplinary research but also indicates potential directions for future research investments and technological developments particularly in the advancement of biomedical imaging technologies.

### 4.1 Limitations

The limitations of this study primarily stem from the constraints of data sources, as it relies solely on the Web of Science Core Collection database, potentially excluding relevant literature from other databases such as PubMed, Medline, or Google Scholar. This restriction may hinder the comprehensiveness of the research findings. Furthermore, bibliometric analysis is dependent on the consistency of keywords and citation information in the literature, which may vary and could impact the accuracy of clustering and co-occurrence analyses. Finally, citation analysis of the literature requires accumulation over time, making it challenging for recently published papers to accumulate an ideal number of citations.

## 5 Conclusion

This study conducts an in-depth analysis of the current status and trends regarding the extravasation of contrast agents in medical imaging examinations using bibliometric methods. Since its initial report in 1950, there has been a marked annual increase in related research literature, particularly after 1979. This trend is closely associated with the rapid advancement of medical imaging technologies. The focus of research is predominantly in the United States, with notable contributions from institutions such as the University of California System and Harvard University, showcasing their significant standing and influence in the international academic community. Through keyword co-occurrence and clustering analysis, the study identified “magnetic resonance imaging,” “computed tomography,” “management,” “diagnosis,” and “contrast agent” as the most frequently occurring keywords, revealing the current research hotspots and future directions. Particularly, the emergence of “gd dtpa” as a burst keyword underscores widespread attention to the safety and efficacy of contrast agents. In addition, the emerging keywords “case report,” “multimodal imaging,” and “leakage” indicate that research on contrast agent extravasation focuses on the analysis of rare cases, the application of multimodal imaging technologies, and an in-depth exploration of the mechanisms and preventive strategies. This reflects the latest developments in the field concerning clinical practice and technological innovation. Future efforts should emphasize precise prevention and management of contrast agent extravasation, optimize care strategies by incorporating individual patient differences, and explore the application of new technologies to reduce extravasation risk through enhanced international collaboration and technological exchange.

## Data Availability

The original contributions presented in the study are included in the article/Supplementary material, further inquiries can be directed to the corresponding author.
