# Supplementary material for: Visual analysis of emerging topics and trends in contrast agent extravasation research in medical imaging: a bibliometric study using CiteSpace and VOSviewer
Source: Front Med (Lausanne). 2025 Feb 19;12:1472637. doi: 10.3389/fmed.2025.1472637 (PMC11879959; doi:10.3389/fmed.2025.1472637)
Supplement: Supplementary file 1 [file Table_1.docx]

**Supplementary Table 1 Literature Search Query**

| **Search Database** | **Search Strategy** |
| --- | --- |
| Web of Science Core Collection | ((TS="extravasat*" OR TS="exosmo*" OR TS="leakage" OR TS="exudat*" OR TS="effus*" OR TS="leaking") AND (TS="contrast agent*" OR TS="contrast medium" OR TS="contrast media" OR (TS=("iodine based contrast media" OR "iodine based contrast medium" OR "iodine based contrast material" OR "iodine contrast media" OR "iodine contrast medium" OR "iodine contrast material" OR "iodine contrast materials" OR "iodine contrast agent" OR "iodine contrast agents" OR "iodinated contrast media" OR "iodinated contrast medium" OR "iodinated contrast material" OR "iodinated contrast materials" OR "iodinated contrast agent" OR "iodinated contrast agents" OR "iodine contrast" OR "iodinated contrast" OR "iodinated radiocontrast" OR (("iodine" NEAR/6 "contrast" NEAR/6 "media") OR ("iodine" NEAR/6 "contrast" NEAR/6 "medium") OR ("iodine" NEAR/6 "contrast" NEAR/6 "material") OR ("iodine" NEAR/6 "contrast" NEAR/6 "materials") OR ("iodine" NEAR/6 "contrast" NEAR/6 "agent") OR ("iodine" NEAR/6 "contrast" NEAR/6 "agents") OR ("iodinated" NEAR/6 "contrast" NEAR/6 "media") OR ("iodinated" NEAR/6 "contrast" NEAR/6 "medium") OR ("iodinated" NEAR/6 "contrast" NEAR/6 "material") OR ("iodinated" NEAR/6 "contrast" NEAR/6 "materials") OR ("iodinated" NEAR/6 "contrast" NEAR/6 "agent") OR ("iodinated" NEAR/6 "contrast" NEAR/6 "agents")) OR "Iohexol" OR "iohexol" OR "Omnipaque" OR "Nycodenz" OR "Exypaque" OR "Compound 545" OR "5-[acetyl(2,3-dihydroxypropyl)amino]-1-N,3-N-bis(2,3-dihydroxypropyl)-2,4,6-triiodobenzene-1,3-dicarboxamide" OR "ioversol" OR "ioversol" OR "optiray" OR "Accupaque" OR "1-N,3-N-bis(2,3-dihydroxypropyl)-5-[(2-hydroxyacetyl)-(2-hydroxyethyl)amino]-2,4,6-triiodobenzene-1,3-dicarboxamide" OR "iodixanol" OR "iodixanol" OR "Visipaque" OR "contrast media 2-5410-3" OR "iodixanol-320" OR "5-[acetyl-[3-[N-acetyl-3,5-bis(2,3-dihydroxypropylcarbamoyl)-2,4,6-triiodoanilino]-2-hydroxypropyl]amino]-1-N,3-N-bis(2,3-dihydroxypropyl)-2,4,6-triiodobenzene-1,3-dicarboxamide" OR "iomeprol" OR "iomeprol" OR "Imeron" OR "Iomeron" OR "1-N,3-N-bis(2,3-dihydroxypropyl)-5-[(2-hydroxyacetyl)-methylamino]-2,4,6-triiodobenzene-1,3-dicarboxamide" OR "iobitridol" OR "iobitridol" OR "5-(3-hydroxy-2-hydroxymethylpropionamido)-N,N'-dimethyl-N,N'-bis(2,3-dihydroxypropyl)-2,4,6-triiodoisophthalamide" OR "1-N,3-N-bis(2,3-dihydroxypropyl)-5-[[3-hydroxy-2-(hydroxymethyl)propanoyl]amino]-2,4,6-triiodo-1-N,3-N-dimethylbenzene-1,3-dicarboxamide" OR "Xenetix" OR "iopromide" OR "iopromide" OR "N,N'-bis(2,3-dihydroxypropyl)-2,4,6-triiodo-5-(2-methoxyacetamido)-N-methylisophthalamide" OR "1-N,3-N-bis(2,3-dihydroxypropyl)-2,4,6-triiodo-5-[(2-methoxyacetyl)amino]-3-N-methylbenzene-1,3-dicarboxamide" OR "iopromid" OR "lopromid" OR "Ultravist" OR "Iopamidol" OR "Iopamidol" OR "Jopamidol" OR "SQ 13,396" OR "Solutrast" OR "Niopam" OR "Isovue" OR "Iopamiro" OR "Gastromiro" OR "B 15,000" OR "B-15000" OR "B15000" OR "B 15000" OR "1-N,3-N-bis(1,3-dihydroxypropan-2-yl)-5-[[(2S)-2-hydroxypropanoyl]amino]-2,4,6-triiodobenzene-1,3-dicarboxamide" OR "iosarcol" OR "iosarcol" OR "N'-(3,5-bis(acetylamino)-2,4,6-triiodobenzoyl)sarcosinyl-N-methylglucamide" OR "Melitrast" OR "Diatrizoate Meglumine" OR "Diatrizoate Meglumine" OR "sodium meglumine diatrizoate" OR "Meglumine Diatrizoate" OR "Diatrizoate Methylglucamine" OR "Methylglucamine Diatrizoate" OR "Amidotrizoate Meglumine" OR "Meglumine Amidotrizoate" OR "sodium;3,5-diacetamido-2,4,6-triiodobenzoate;3,5-diacetamido-2,4,6-triiodobenzoic acid;(2R,3R,4R,5S)-6-(methylamino)hexane-1,2,3,4,5-pentol " OR "Sinografin" OR "Urografin" OR "Amidotricoic Acid" OR "Diatrizoic Acid Methylglucamine" OR "Verografin" OR "Urovist" OR "Triombrin" OR "Triombrast" OR "Renografin" OR "Renograffin" OR "Reno MDip" OR "Reno M Dip" OR "Reno 60" OR "Angiografin" OR "Amidotrizoic Acid" OR "Gastrograffin" OR "Gastrographin" OR "Gastrografin" OR "meglumine ioxithalamate" OR "meglumine ioxithalamate" OR "meglumine ioxitalamate" OR "meglumine ioxythalamate" OR "methylglucamine ioxitalamate" OR "methylglucamine ioxithalamate" OR "Telebrix 350" OR "Telebrix Gastro" OR "3-acetamido-5-(2-hydroxyethylcarbamoyl)-2,4,6-triiodobenzoic acid;(2R,3R,4R,5S)-6-(methylamino)hexane-1,2,3,4,5-pentol" OR "gadolinium" OR "gadolinium based contrast media" OR "gadolinium based contrast medium" OR "gadolinium based contrast material" OR "gadolinium contrast media" OR "gadolinium contrast medium" OR "gadolinium contrast material" OR "gadolinium contrast agent" OR "gadolinium contrast agents" OR "gd based contrast media" OR "gd based contrast medium" OR "gd contrast media" OR "gd contrast medium" OR "gd contrast material" OR "gd contrast agent" OR "gd contrast agents" OR (("gadolinium" NEAR/6 "contrast" NEAR/6 "media") OR ("gadolinium" NEAR/6 "contrast" NEAR/6 "medium") OR ("gadolinium" NEAR/6 "contrast" NEAR/6 "material") OR ("gadolinium" NEAR/6 "contrast" NEAR/6 "materials") OR ("gadolinium" NEAR/6 "contrast" NEAR/6 "agent") OR ("gadolinium" NEAR/6 "contrast" NEAR/6 "agents") OR ("gd" NEAR/6 "based" NEAR/6 "contrast" NEAR/6 "media") OR ("gd" NEAR/6 "based" NEAR/6 "contrast" NEAR/6 "medium") OR ("gd" NEAR/6 "contrast" NEAR/6 "media") OR ("gd" NEAR/6 "contrast" NEAR/6 "medium") OR ("gd" NEAR/6 "contrast" NEAR/6 "material") OR ("gd" NEAR/6 "contrast" NEAR/6 "agent") OR ("gd" NEAR/6 "contrast" NEAR/6 "agents")) OR "gadoterate meglumine" OR "gadoterate meglumine" OR "gadoterate" OR "Dotarem" OR "Guerbet" OR "Gadolinium-DOTA" OR "Gd-DOTA" OR "2-[4,7-bis(carboxylatomethyl)-10-(carboxymethyl)-1,4,7,10-tetrazacyclododec-1-yl]acetate;gadolinium(3+);(2R,3R,4R,5S)-6-(methylamino)hexane-1,2,3,4,5-pentol" OR "gadobutrol" OR "Gadobutrol" OR "Gadovist" OR " Gd-DO3A-butriol" OR "Gadolinium-DO3A-butriol" OR "Gd-BT-DO3A" OR "2-[4,10-bis(carboxylatomethyl)-7-[(2R,3S)-1,3,4-trihydroxybutan-2-yl]-1,4,7,10-tetrazacyclododec-1-yl]acetate;gadolinium(3+)" OR "gadoteridol" OR "Gadoteridol" OR "ProHance" OR "gadolinium 1,4,7-triscarboxymethyl-1,4,7,10-tetraazacyclododecane" OR "Gd-HP-D03A" OR "Gd-hydroxypropyl-D03A" OR "gadolinium 1,4,7-tris(carboxymethyl)-10-(2'-hydroxypropyl)-1,4,7,10-tetraazacyclododecane" OR "gadolinium HP-DO3A" OR "Gd-HPDO3A" OR "GdHPDO3A" OR "Gd-HP-DO3A" OR "Gd DO3A" OR "SQ 32692" OR "SQ 32,692" OR "Gadolinium-hydroxypropyl-DO3A" OR "2-[4,7-bis(carboxylatomethyl)-10-(2-hydroxypropyl)-1,4,7,10-tetrazacyclododec-1-yl]acetate;gadolinium(3+)" OR "gadobenic acid" OR "gadobenic acid" OR "Gadobenate dimeglumine" OR "3,6,9-triaza-12-oxa-3,6,9-tricarboxymethylene-10-carboxy-13-phenyltridecanoic acid" OR "Gd-BOPTA" OR "B 19036" OR "MultiHance" OR "gadobenate dimeglumine" OR "gadobenate" OR "gadolinium-BOPTA-Dimeg" OR "Gd(BOPTA)2" OR "gadolinium-benzyloxypropionyl tetraacetate" OR "Gadolinium-BOPTA" OR "2-[2-[2-[bis(carboxylatomethyl)amino]ethyl-(carboxylatomethyl)amino]ethyl-(carboxylatomethyl)amino]-3-phenylmethoxypropanoate;gadolinium(3+);hydron;(2R,3R,4R,5S)-6-(methylamino)hexane-1,2,3,4,5-pentol" OR "gadolinium ethoxybenzyl DTPA" OR "gadolinium ethoxybenzyl DTPA" OR "Gadoxetate disodium" OR "Primovist" OR "Gd-EOB-DTPA" OR "gadolinium ethoxybenzyl diethylenetriaminepentaacetic acid" OR "gadoxetic acid disodium" OR "gadolinium (4S)-4-(4-ethoxybenzyl)-3,6,9-tris(carboxylatomethyl)-3,6,9-triazaundecanoic acid disodium salt" OR "gadoxetate disodium" OR "disodium gadoxetate" OR "gadoxetate" OR "Eovist" OR "gadoxetic acid" OR "Gadolinium-ethoxybenzyl-DTPA" OR "2-[[2-[bis(carboxylatomethyl)amino]-3-(4-ethoxyphenyl)propyl]-[2-[bis(carboxylatomethyl)amino]ethyl]amino]acetate;gadolinium(3+)" OR "gadopiclenol" OR "Gadopiclenol" OR "Elucirem" OR "2-[3,9-bis[1-carboxylato-4-(2,3-dihydroxypropylamino)-4-oxobutyl]-3,6,9,15-tetrazabicyclo[9.3.1]pentadeca-1(15),11,13-trien-6-yl]-5-(2,3-dihydroxypropylamino)-5-oxopentanoate;gadolinium(3+)" OR "Gadolinium 2,2',2''-(3,6,9,15-tetraazabicyclo(9.3.1)pentadeca-1(15),11,13-triene-3,6,9-triyl)tris(5-((2,3-dihydroxypropyl)amino)-5-oxopentanoate)" OR "3,6,9,15-Tetraazabicyclo(9.3.1)pentadeca-1(15),11,13-triene-3,6,9-triacetic acid, alpha, alpha', alpha''-tris(3-((2,3-dihydroxypropyl)amino)-3-oxopropyl)-, gadolinium salt (1:1)" OR "gadodiamide" OR "Gadodiamide" OR "Omniscan" OR "Gd-DTPA bis-(methylamide)" OR "Gd-DTPA-BMA" OR "Gadolinium-BMA-DTPA" OR "2-[bis[2-[carboxylatomethyl-[2-(methylamino)-2-oxoethyl]amino]ethyl]amino]acetate;gadolinium(3+)" OR "gadoversetamide" OR "Gadoversetamide" OR "Optimark" OR "Gadolinium-DHEA-BMA" OR "2-[bis[2-[carboxylatomethyl-[2-(2-methoxyethylamino)-2-oxoethyl]amino]ethyl]amino]acetate;gadolinium(3+)" OR "Gadolinium DTPA" OR "Gadopentetate dimeglumine" OR "Magnevist" OR "Gadolinium-DTPA" OR "Gd-DTPA" OR "Gadolinium Diethylenetriaminepenta-acetic Acid" OR "Gadolinium Diethylenetriaminepenta acetic Acid" OR "Gadopentetic Acid" OR "Gadopentetate" OR "Gadolinium DTPA Dimeglumine" OR "Gadolinium DTPA Disodium" OR "Magnograf" OR "2-[bis[2-carboxylatomethyl(carboxymethyl)amino]ethyl]amino]acetate;gadolinium(3+); (2R,3R,4R,5S)-6-(methylamino)hexane-1,2,3,4,5-pentol" OR "contrast media" OR "contrast medium" OR "contrast material" OR "contrast materials" OR "contrast agent" OR "contrast agents" OR "radiocontrast media" OR "radiocontrast medium" OR "radiocontrast material" OR "radiocontrast materials" OR "radiocontrast agent" OR "radiocontrast agents" OR "contrast induced" OR "contrast related" OR "contrast exposure" OR "contrast dosage" OR "contrast dose" OR "contrast doses" OR "contrast enhanced" OR "contrast administration" OR "3-hydroxyephedrine" OR "3-iodo-2-hydroxy-6-methoxy-N-((1-ethyl-2-pyrrolidinyl)methyl)benzamide" OR "7-(6-fluoropyridin-3-yl)-5H-pyrido(4,3-b)indole" OR "Acetrizoic Acid" OR "albumin-(gadolinium-DTPA)" OR "Albunex" OR "altanserin" OR "barium" OR "barium fluoride" OR "Barium Sulfate" OR "BR14 contrast agent" OR "BY 963" OR "C.I. Fluorescent Brightening Agent 28" OR "diethylenetriamine pentaacetic acid dianhydride" OR "epidepride" OR "Ethiodized Oil" OR "ferrioxamine B" OR "ferumoxides" OR "ferumoxtran-10" OR "Fluorescein" OR "flutemetamol" OR "FS 069" OR "gadofosveset trisodium" OR "gadolinium 1,4,7,10-tetraazacyclododecane-N,N',N'',N'''-tetraacetate" OR "gadolinium chloride" OR "gadoterate meglumine" OR "glucose, glycerol, hydroxyethyl starch, perfluorodecalin, perfluorotripropylamine, pluronic F-68, salts, yolk phospholipids drug combination" OR "iobenzamic acid" OR "iocarmate meglumine" OR "Iodamide" OR "Iodipamide" OR "Iodized Oil" OR "Iodohippuric Acid" OR "Iodopyracet" OR "iodoxamic acid" OR "Ioglycamic Acid" OR "Iopanoic Acid" OR "iopentol" OR "Iophendylate" OR "Iothalamate Meglumine" OR "Iothalamic Acid" OR "iotrolan" OR "Ioxaglic Acid" OR "ioxilan" OR "ioxitalamic acid" OR "Ipodate" OR "Magnetite Nanoparticles" OR "manganese(III)tetraphenylporphine sulfonate" OR "Meglumine" OR "meglumine iodipamide" OR "meglumine iotroxinate" OR "Metrizamide" OR "Metrizoic Acid" OR "Mn-PyC3A" OR "motexafin gadolinium" OR "N,N'-bis(pyridoxal-5-phosphate)ethylenediamine-N,N'-diacetic acid" OR "oxypolygelatine" OR "perflubron" OR "perfluoropentane" OR "perfluorophenanthrene" OR "perflutren" OR "poly-gamma-benzyl-L-glutamate" OR "polylysine-(Gd-DTPA)" OR "Propyliodone" OR "setoperone" OR "SHU 454" OR "SHU 508" OR "Sonazoid" OR "sprodiamide" OR "tetraphenylporphine sulfonate" OR "tozuleristide" OR "Tyropanoate" OR "urovision" OR "3-hydroxyephedrine" OR "3-iodo-2-hydroxy-6-methoxy-N-((1-ethyl-2-pyrrolidinyl)methyl)benzamide" OR "7-(6-fluoropyridin-3-yl)-5H-pyrido(4,3-b)indole" OR "Acetrizoic Acid" OR "albumin-(gadolinium-DTPA)" OR "Albunex" OR "altanserin" OR "barium fluoride" OR "Barium Sulfate" OR "BR14 contrast agent" OR "BY 963" OR "C.I. Fluorescent Brightening Agent 28" OR "diethylenetriamine pentaacetic acid dianhydride" OR "epidepride" OR "Ethiodized Oil" OR "ferrioxamine B" OR "ferumoxides" OR "ferumoxtran-10" OR "Fluorescein" OR "flutemetamol" OR "FS 069" OR "gadofosveset trisodium" OR "gadolinium 1,4,7,10-tetraazacyclododecane-N,N',N'',N'''-tetraacetate" OR "gadolinium chloride" OR "gadoterate meglumine" OR "glucose, glycerol, hydroxyethyl starch, perfluorodecalin, perfluorotripropylamine, pluronic F-68, salts, yolk phospholipids drug combination" OR "iobenzamic acid" OR "iocarmate meglumine" OR "Iodamide" OR "Iodipamide" OR "Iodized Oil" OR "Iodohippuric Acid" OR "Iodopyracet" OR "iodoxamic acid" OR "Ioglycamic Acid" OR "Iopanoic Acid" OR "iopentol" OR "Iophendylate" OR "Iothalamate Meglumine" OR "Iothalamic Acid" OR "iotrolan" OR "Ioxaglic Acid" OR "ioxilan" OR "ioxitalamic acid" OR "Ipodate" OR "Magnetite Nanoparticles" OR "manganese(III)tetraphenylporphine sulfonate" OR "Meglumine" OR "meglumine iodipamide" OR "meglumine iotroxinate" OR "Metrizamide" OR "Metrizoic Acid" OR "Mn-PyC3A" OR "motexafin gadolinium" OR "N,N'-bis(pyridoxal-5-phosphate)ethylenediamine-N,N'-diacetic acid" OR "oxypolygelatine" OR "perflubron" OR "perfluoropentane" OR "perfluorophenanthrene" OR "perflutren" OR "poly-gamma-benzyl-L-glutamate" OR "polylysine-(Gd-DTPA)" OR "Propyliodone" OR "setoperone" OR "SHU 454" OR "SHU 508" OR "Sonazoid" OR "sprodiamide" OR "tetraphenylporphine sulfonate" OR "tozuleristide" OR "Tyropanoate" OR "urovision") AND (TS=imaging OR TS=imaging examination* OR TS=imaging stud* OR TS=imageology OR TS=imageological OR TS= radiol* OR WC=(Radiology, Nuclear Medicine & Medical Imaging))))) |
